# One-pot MCDA-CRISPR-Cas-based detection platform for point-of-care testing of severe acute respiratory syndrome coronavirus 2

**DOI:** 10.3389/fmicb.2024.1503356

**Published:** 2024-12-06

**Authors:** Xiaoxia Wang, Rui Yang, Tian Tang, Yuzhen Zhou, Heng Chen, Yihao Jiang, Shirong Zhang, Sihan Qin, Meijuan Wang, Chuan Wang

**Affiliations:** ^1^West China School of Public Health and West China Fourth Hospital, Sichuan University, Chengdu, Sichuan, China; ^2^Central & Clinical Laboratory of Sanya People’s Hospital, Sanya, Hainan, China; ^3^Chengdu Center for Disease Control and Prevention, Chengdu, Sichuan, China

**Keywords:** COVID-19, SARS-CoV-2, MCTOP, Cas12b, point-of-care testing

## Abstract

Compared to quantitative real-time PCR (q-PCR), CRISPR-Cas-mediated technology is more suitable for point-of-care testing (POCT) and has potential for wider application in the future. Generally, the operational procedure of CRISPR-Cas-mediated diagnostic method consists of two independent steps, the reaction of signal amplification and the CRISPR-Cas-mediated signal detection. Complex multi-step procedures can easily lead to cross-contamination. To develop a convenient and rapid method for severe acute respiratory syndrome coronavirus 2 (SARS-CoV-2) detection, we propose a MCTOP method (*M*ultiple cross displacement amplification-*C*RISPR-Cas12b-based *t*esting in *o*ne-*p*ot), which targets the open reading frame 1ab (ORF1ab) and nucleocapsid protein (N) gene of SARS-CoV-2. This method combines MCDA isothermal amplification and CRISPR-Cas-mediated sequence-specific detection into a one-pot reaction. The optimal reaction was achieved with isothermal amplification of 40 min and CRISPR-Cas-based detection of 15 min, both at 64°C. Then, the results can be visualized by the real-time fluorescence instrument and also lateral flow biosensor. The lowest detection limit of the proposed method is 10 copies of each of target sequences, and it has no cross-reactivity with non-SARS-CoV-2 templates. In a clinical test of 70 pharyngeal swab samples, MCTOP assay showed a specificity of 100% and sensitivities of 98 and 96% for the real-time fluorescence instrument and lateral flow biosensor, respectively. The MCTOP developed in this study is a rapid, convenient, highly sensitive, and specific method for SARS-CoV-2 nucleic acid detection. It can be used as an effective point-of-care testing (POCT) tool for clinical diagnosis and epidemiologic surveillance of SARS-CoV-2 infections, especially suitable for the basic, field and clinical laboratory.

## Introduction

The emerging disease caused by SARS-CoV-2 is named as coronavirus disease 2019 (COVID-19), which was classified as a pandemic by the World Health Organization (WHO) in March 2020 due to its highly contagious nature. This pandemic presented an unprecedented challenge to global public health systems (Won et al., 2020; Zhu et al., 2020; Wang et al., 2023). At present, many countries have discontinued COVID-19 reporting and integrated it into longer-term disease management. But COVID-19 remains a potential public health concern, particularly in remote areas with limited access to laboratory testing and at high-traffic border crossings. Hence, early diagnosis of patients infected with SARS-CoV-2 has become a top priority, with fast and accurate identification of pathogens playing a pivotal role in sustaining early warning, surveillance, reporting, variant tracking, and early clinical care provision. In particular, point-of-care testing (POCT) does not require complex experimental instruments or special laboratory sites, enabling rapid, low-cost, and portable detection of the SARS-CoV-2. It can also address the need for fast and convenient testing in remote areas, border ports, and at home. Nowadays, the reverse transcription quantitative real-time polymerase chain reaction (RT-qPCR) targeting ORF1ab, N, and/or E genes is widely used and has played a significant role in detection of SARS-CoV-2 (Rahbari et al., 2021). But this method has some disadvantages, including complex operation, time consumption, and the requirement for specialized equipment and skilled personnel; therefore, it is not suitable for POCT (Chu et al., 2020). Hence, it remains critical requirement to develop simpler, easy-to-handle, and faster methods for diagnosing COVID-19.

At present, prokaryotic clustered regularly interspaced short palindromic repeats and clustered regularly interspaced short palindromic repeats-associated protein (CRISPR-Cas) immune system is anticipated to provide a powerful method to overcome above problem and has immense prospects for biotechnological applications as a new generation of molecular diagnostic approaches (Chertow, 2018). A variety of Cas effectors, including Cas12a, Cas12b, Cas13a, and Cas14, have been shown to have cleavage activity on single-strand nucleic acids, which has made the development of detection methods with highly specific and ultra-sensitive possible (Li et al., 2018; Li et al., 2019). In comparison with the conventional PCR methods, CRISPR-Cas-based tests do not require the precision thermal cycler for thermal change and have high amplification efficiency, as they are less time-consuming in isothermal reaction. Up to now, CRISPR-Cas-based assays have been developed for the detection of multiple viruses, including CRISPR-Cas12a/Cas14-based DNA Endonuclease Targeted CRISPR Tans Reporter (DETECTR) assays used for human papillomavirus (HPV) diagnosis (Harrington et al., 2018), and a CRISPR-Cas13a-based Specific High-Sensitivity Enzymatic Reporter Unlocking (SHERLOCK) assay used for Dengue virus (DENV), Zika virus (ZIKV), and HPV detection (Gootenberg et al., 2017). The detection principle of CRISPR-Cas-based methods consists of two main steps, including signal amplification by PCR or isothermal amplification, followed by the detection of amplified signals through CRISPR-Cas-based collateral reporter decoding. In the actual operational process, most CRISPR-Cas-based methods include two independent reaction steps, a multitude of manual procedures, and liquid handling, which limit their widespread application, especially in grassroots medical institutions, due to complex multi-step procedures and high risk of potential cross-contamination (Broughton et al., 2020; Huang et al., 2020; Zhu et al., 2021). At present, some researchers have developed several one-pot methods for SARS-CoV-2 detection. Although these methods offer advantages such as high sensitivity, accuracy, and stability, they rely on protospacer adjacent motifs (PAMs) sites, which limit the selection flexibility of target sites (Ding et al., 2020; Guo et al., 2020; Chen et al., 2022). CRISPR-top (*CRISPR*-mediated *t*esting in *o*ne-*p*ot) is a one-pot, CRISPR-Cas-based nucleic acid detection platform. Specifically, a PAM site is introduced to the target sequence by an engineered primer, thereby expanding the application potential of this technique (Li et al., 2021; Qiu et al., 2022; Qiu et al., 2023).

In this study, we combined multiple cross displacement amplification (MCDA) (Wang et al., 2015) with CRISPRR-Cas12b-based detection to develop a novel method called MCTOP, which performs both the signal amplification and CRISPR-Cas-mediated detection of amplified signals steps in one-pot reaction tube for rapid detection of SARS-CoV-2, targeting the ORF1ab and N genes. Briefly, the MCTOP reaction is carried out at a constant temperature, and the test results can be visualized using a real-time fluorescence instrument or a lateral flow biosensor. Notably, based on the latter detection method, a field detection scheme can be developed. Additionally, target genes lacking PAMs site are still detectable by MCTOP assay when engineering primers are modified to contain a PAM site.

## Materials and methods

### Materials and reagents

Synthetic MCDA primers and reporter molecules (single-strand DNA; ssDNA) were purchased from Sangon Biotech Co., Ltd. (Shanghai, China) and guide RNAs (gRNAs) were obtained from GeneScript Biotech Co, Ltd. (Beijing, China). Plasmids containing the target sequences of ORF1ab and N genes from each virus genome (SARS-CoV-2, SARS-CoV, and MERS-CoV) were synthesized by Sangon Biotech Co., Ltd. (Shanghai, China). Bst 3.0 WarmStart polymerase, Deoxynucleotide (dNTP) Solution Mix, and WarmStart RTx Reverse Transcriptase were purchased from New England Biolabs. AapCas12b (C2c1) and lateral flow biosensor were obtained from EZassay Biotech Co., Ltd. (Shenzhen, China).

### Primer and gRNA design

The primers for target genes, ORF1ab and N genes of SARS-CoV-2 (GenBank MN908947, Wuhan-Hu-1), were designed by online primer design software version 4.0^1^ (Eiken Chemical Co., Ltd., Tokyo, Japan) according to the principles of MCDA and MCTOP. Subsequently, primer specificity was checked using the Primer-Blast tool.^2^ The primers were evaluated for the formation of secondary structures and/or primer dimers using OligoAnalyzer online software version 3.1 (Integrated DNA Technologies, Coralville, IA). The design of CRISPR gRNAs was performed in accordance with the MCTOP rules. The sequences of primer and gRNAs used in this study are provided in Table 1.

**Table 1:** The primers, reporter molecule and gRNAs used in this study.

Primers/gRNAs ^a^	Sequences and modifications ^b^	Length (nt) ^c^	Genes
ORF1ab-F1	5’-TGCATCGTGTTGTCTGT-3’	17	*ORF1ab*
ORF1ab-F2	5’-TTACACCGCAAACCCGT-3’	17	
ORF1ab-CP1	5′- AGCACAAGTTGTAGGTATTTGTACATTCCCGTTGCCACATAGATCA −3’	46	
ORF1ab-CP2	5′- CGTCTGCGGTATGTGGAAAGATTGTGCATCAGCTGACTG −3’	40	
ORF1ab-C1	5’-AGCACAAGTTGTAGGTATTTGTACA-3’	25	
ORF1ab-C2	5’-CGTCTGCGGTATGTGGAAAG-3’	20	
ORF1ab-D1	5’-CACAAAATCCTTTAGGATTTG-3’	21	
ORF1ab-D2	5’-CTGTAGTTGTGATCAACTC-3’	19	
ORF1ab-R1	5’-AAAACCCACAGGGTCAT-3’	17	
ORF1ab-R2	5’-CTTAAAAACACAGTCTGTAC-3’	20	
ORF1ab-gRNA	5’-GUCUAGAGGACAGAAUUUUUCAACGGGUGUGCCAAUGGCCACUUUCCAGGUGGCAAAGCCCGUUGAGCUUCUCAAAUCUGAGAAGUGGCACCCGUUGCCACAUAGAUCAUC-3’	111	
N-F1	5’-ATTGAACCAGCTTGAGAGCA-3’	20	*N*
N-F2	5’-GCAATTTGCGGCCAATGT-3’	18	
N-CP1	5’-TTGCCGAGGCTTCTTAGAAGCCAGGCCAACAACAACAAGGC-3’	41	
N-CP2	5’-AACACAAGCTTTCGGCAGACGTTGATTAGTTCCTGGTCCCCA-3’	42	
N-C1	5’-TTGCCGAGGCTTCTTAGAAGCC-3’	22	
N-C2	5’-AACACAAGCTTTCGGCAGACGT-3’	22	
N-D1	5’-CAGCAGCAGATTTCTTAGTGACAG-3’	24	
N-D2	5’-GGTCCAGAACAAACCCAAGG-3’	20	
N-R1	5’-TTTCATTGTATGCTTTAGTGGCA-3’	23	
N-gRNA	5’-GUCUAGAGGACAGAAUUUUUCAACGGGUGUGCCAAUGGCCACUUUCCAGGUGGCAAAGCCCGUUGAGCUUCUCAAAUCUGAGAAGUGGCACCAUUGUAUGCUUUAGUGGCA-3’	111	
Fluorescent reporter	5’-FAM-TTATTAT-BHQ1-3’	7	
Lateral flow reporter	5’-FAM-TTATTAT-Biotin-3’	7	

a ORF1ab, open reading frame 1a/b; N, nucleoprotein gene.

b ORF1ab-CP1 and N-R1primers were modified in linker region with PAM site (TTC and TTT marked in red).

c nt, nucleitide.

### MCTOP reaction

The MCTOP reaction system contained following components: isothermal amplification buffer (1×), 1.4 mM dNTPs, 8 mM MgSO_4_, 0.4 μM each of F1 and F2, 0.8 μM each of C1, C2, R1, R2, D1 and D2, 1.6 μM each of CP1 and CP2, 0.32 U/μL Bst 3.0 DNA polymerase, 0.3 U/μL WarmStart RTx Reverse Transcriptase, 600 nM fluorescent probe for RT-PCR assay (200 nM fluorescent probe for lateral flow assay), and 1 μL templates. Total reaction volume was adjusted to 25 μL with double distilled water. A blank control (1 μL sterile water instead of templates) was included in each batch. Then, 10 μL of CRISPR/Cas-based detection reagent, 500 nM CRISPR associated protein 12b (AapCas12b) nuclease and 500 nM gRNA in 1× Cas12b reaction buffer, were added to the tube caps. The MCTOP reactions were incubated at 64°C for 40 min. After incubation, the tubes were spun down to mix the CRISPR/Cas-based detection reagent with the MCDA reaction, and the reaction tubes were incubated continuously at 64°C for 15 min for further AapCas12b based collateral activity. For the lateral flow-based detection, we combined the two duplicate reaction mixes into one tube and carried out lateral flow detection.

### Evaluating the sensitivity and specificity of COVID-19 MCTOP assay

The construction of ORF1ab- and N-plasmid was performed by inserting the synthetic ORF1ab and N gene fragments into the plasmids, respectively. The ORF1ab- and N-plasmid were used for sensitivity and optimal amplification analysis of COVID-19 MCTOP assay. In the sensitivity analysis of the COVID-19 MCTOP assay, the ORF1ab- and N-plasmid were 10-fold serially diluted to different concentrations, ranging from 1 × 10^4^ copies/μL to 1 × 10^0^ copies/μL. Next, 1 microliter of each dilution was used as a template for MCTOP amplification to determine the lowest limit of detection for lateral flow biosensor and the real-time fluorescence instrument (QuantStudio 3, Applied Biosystems), respectively. The ORF1ab- and N-plasmid of SARS-CoV and MERS-CoV, which contain the corresponding synthetic ORF1ab and N gene fragments, were used to validate the specificity of COVID-19 MCTOP assay.

### The common inhibitors tolerance test

Given the potential impact of inhibitors on the amplification reaction, we evaluated the influence of three common inhibitors, sodium dodecyl sulfate (SDS), ethylenediaminetetraacetic acid (EDTA), and ethanol, on COVID-19 MCTOP assay. SDS is an ionic detergent commonly used in sample analysis, and has been reported as a DNA polymerase inhibitor (Schrader et al., 2012). Similarly, EDTA, which chelates metal ions (such as Mg^2+^), and ethanol, which is often used in nucleic acid purification, have been found to inhibit the amplification reaction. To test the tolerance of our method to these inhibitors, 0.01, 0.001 and 0.0001% of SDS, 10 mM, 1 mM and 0.1 mM of EDTA, or 5 and 10% of ethanol were added into the MCTOP reactions, and the results were detected by real-time fluorescence instrument.

### Applicability of COVID-19 MCTOP assay to clinical samples

Seventy pharyngeal swab samples, including 50 SARS-CoV-2 nucleic acid positive samples confirmed by RT-qPCR at Chengdu Center for Disease Control and Prevention in Sichuan, and 20 negative samples from Sanya People’s Hospital in Hainan, were collected as test samples to verify the clinical application of COVID-19 MCTOP assay. We used the nucleic acid rapid extraction kit from BioPerfectus Technologies Co., Ltd. for the rapid extraction of RNA from clinical samples. Aliquots of 1 μL of RNA templates were used for both RT-qPCR and COVID-19 MCTOP assays, respectively.

## Results

### MCTOP design

The MCTOP reaction combines MCDA-mediated isothermal amplification with CRISPR/Cas-based detection, enabling one-pot nucleic acid detection at a single uniform temperature. A PAM site modification unrelated to the target sequence is designed in the connected region or 5′ ends of CP1 (cross primer 1) or R1 (amplification primer R1) primers. During amplification, the MCDA-amplified products will be labeled with PAM sites, which can be recognized by the corresponding Cas12b/gRNA system. If the target regions closely followed by a PAM site are complementary to the gRNA, Cas12b will be activated and will trans-cleavage the reporter molecule (single-strand DNA; ssDNA). Of note, if the target genes satisfy the requirement of MCDA design, any target genes can be detected by the MCTOP assay as long as the cross primer or amplification primers are PAM-modified, even target genes have no suitable PAM sites for CRISPR-Cas12b/gRNA complex. When the result is reported as fluorescence units, the total detection time is 70 min, including nucleic acid preparation of 15 min and MCTOP reaction of 55 min. When the results are reported by lateral flow biosensor, extra 5 min for result interpretation is needed.

### COVID-19 MCTOP test

**Figure 1:**
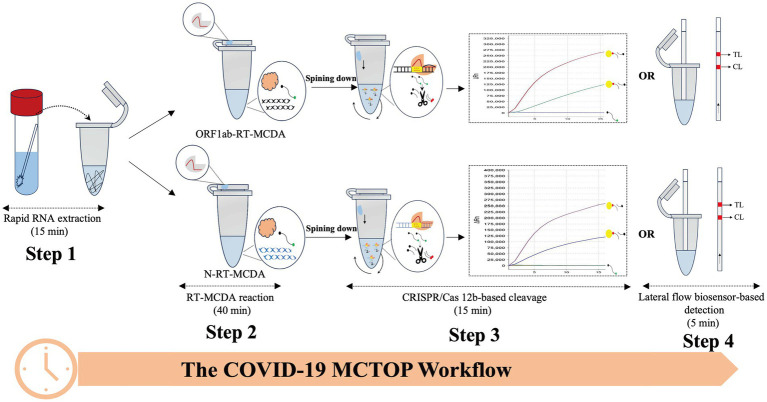
The workflow diagram of COVID-19 MCTOP assay The COVID-19 MCTOP detection consists of four stages: Step 1 is rapid nucleic acid preparation of 15 min, the second step is RT-MCDA reaction of 40 min, and in the third step, Cas12b-gRNA complex is centrifuged into the RT-MCDA reaction mix for 15 min of target cleavage and CRISPR/Cas-based detection. Cleavage of reporter molecule (ssDNA) can be reported by real-time fluorescence (Step 3) and lateral flow biosensor of 5 min (Step 4).

In order to establish a reliable MCTOP assay for the detection of SARS-CoV-2, named COVID-19 MCTOP, two sets of primers were designed to target ORF1ab and N genes of SARS-CoV-2, respectively. The linker regions of ORF1ab-CP1 and N-R1 primers were modified with PAM sites (TTC and TTT) (Table 1) to satisfy the requirements of MCTOP. In a single tube, multiple reactions were conducted at a constant temperature of 64°C, including reverse transcription of RNA templates, multiple cross displacement amplification, and detection of amplified signals based on CRISPR-Cas12b. Briefly, following the extraction of SARS-CoV-2 RNA (Figure 1, Step 1), the cDNA generated from the reverse transcription reaction using reverse transcriptase was used as the template for subsequent isothermal amplification (Figure 1, Step 2). During MCTOP reaction, PAM site from ORF1ab-CP1 and N-R1 primers was inserted in frame into ORF1ab- and N-MCDA products to attract Cas12b/gRNA complex, allowing it to hybridize with amplification product. This hybridization triggered the trans-cleavage activation of Cas12b, which cleaved the ssDNA reporter molecules (Figure 1, Step 3). The test results were visualized by the real-time fluorescence instrument (Figure 1, Step 3) or lateral flow biosensor (Figure 1, Step 4). Therefore, total time for real-time COIVD-19 MCTOP reaction was estimated to be 70 min, including 15 min for nucleic acid preparation and 55 min for MCTOP reaction. Lateral flow COVID-19 MCTOP can be completed also within 75 min, even with the addition of a result interpretation step (5 min) in addition to the aforementioned steps.

### The principle of biosensor for visualization of MCTOP results

**Figure 2:**
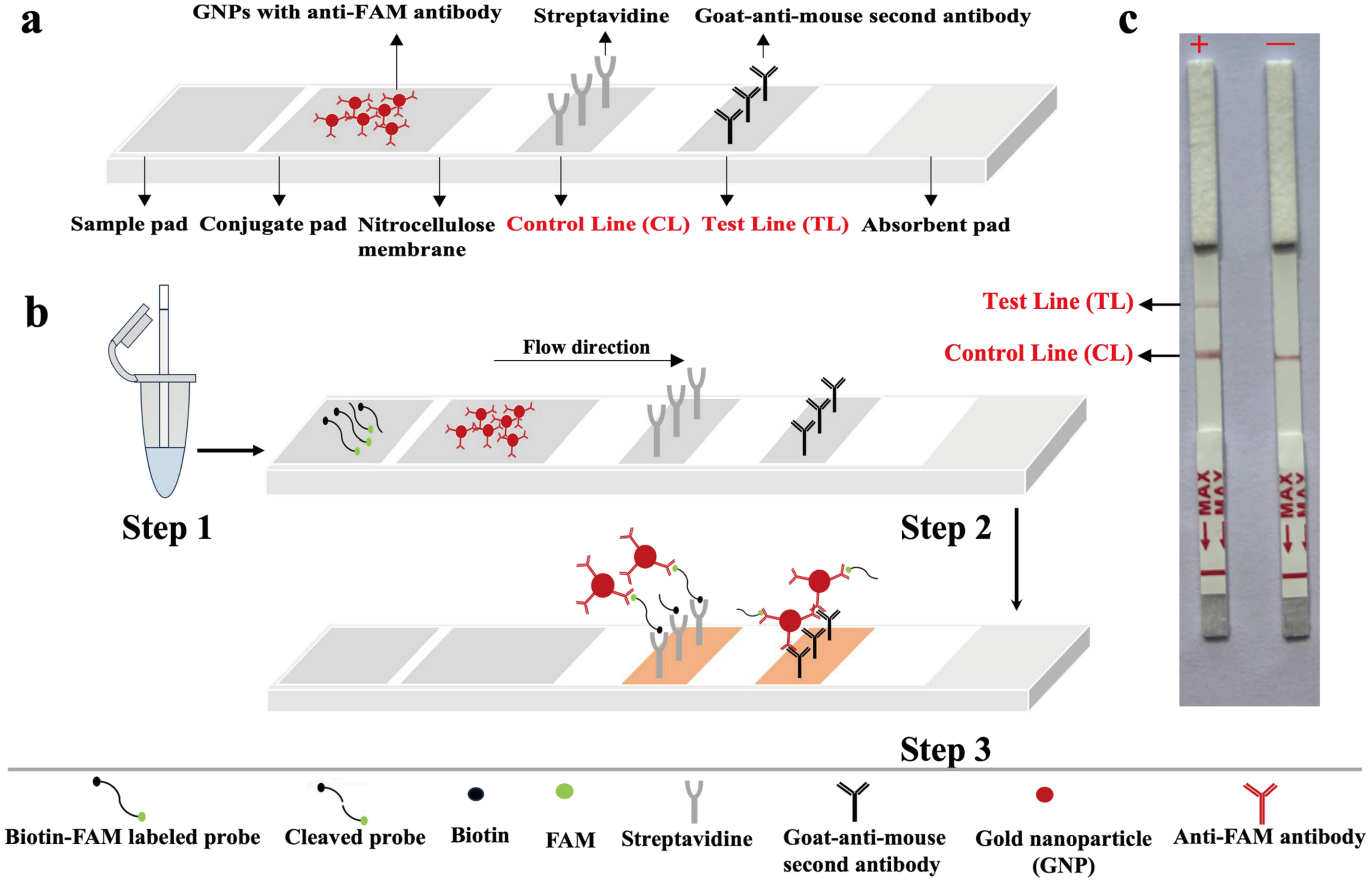
The principle for visualization of MCTOP products by biosensor. **(A)** The details of biosensor design. **(B)** The schematic of biosensor for MCTOP products visualization. **(C)** Schematic illustration of the MCTOP results interpretation. +, results of positive MCTOP test (clearly visible red lines for both control line (CL) and test line (TL) were present on the biosensor); −, results of negative MCTOP test (only the control line (CL) was present on the biosensor).

As presented in Figure 2A, first, inserts the lateral flow strip into the reaction tube (Figure 2B, Step 1). Afterwards, the detection mixture moves forward on the biosensor by capillary action to rehydrate functional nanoparticles (anti-FAM antibodies-GNPs) in conjugate region. As the movement continues, the reporter molecules end-labeled with FAM molecules, are combined with anti-FAM antibodies-GNPs for chromogenic visualization. The streptavidin coated on the control line zone captures reporter molecules that are end-labeled with biotin, resulting in the visualization of the control line (Figure 2B, Step 2). If the reporter molecules have been cleaved by the activated Cas12b effector, the biotin-labeled end and FAM-labeled end will be separated. In this case, goat anti-mouse secondary antibodies coated on the test line zone can capture anti-FAM antibodies-GNPs complex of reporter molecule, leading to the visualization of the test line. For positive amplifications, both the control line (CL) and test line (TL) are observed simultaneously in biosensor, whereas for negative amplifications, only the control line (CL) is observed (Figure 2B, Step 3 and Figure 2C).

### Optimal conditions for MCTOP assay

**Figure 3:**
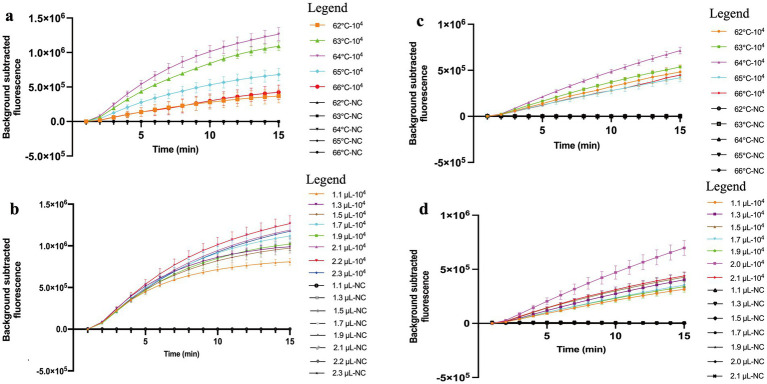
Optimal reaction conditions of the COVID-19 MCTOP assay. Error bars represent the means ± standard error of means (SEM) from three replicates. **(A)** Optimal reaction temperature for ORF1ab gene detection. The reactions were performed under temperatures ranging from 62°C to 66°C at 1°C intervals. **(B)** Optimal primer concentration for ORF1ab gene detection. Serial volumes of MCDA primer premixture (2.2 μL and 1.1 to 2.3 μL with 0.2 μL intervals) were used to prepare the reaction mixtures. Primer concentrations for serial volumes were as follows. 1.1 μL (0.2 μM each of F1 and F2, 0.4 μM each of C1, C2, R1, R2, D1 and D2, 0.8 μM each of CP1 and CP2), 1.3 μL (0.24 μM each of F1 and F2, 0.47 μM each of C1, C2, R1, R2, D1 and D2, 0.95 μM each of CP1 and CP2), 1.5 μL (0.27 μM each of F1 and F2, 0.55 μM each of C1, C2, R1, R2, D1 and D2, 1.09 μM each of CP1 and CP2), 1.7 μL (0.31 μM each of F1 and F2, 0.62 μM each of C1, C2, R1, R2, D1 and D2, 1.24 μM each of CP1 and CP2), 1.9 μL (0.35 μM each of F1 and F2, 0.69 μM each of C1, C2, R1, R2, D1 and D2, 1.38 μM each of CP1 and CP2), 2.1 μL (0.38 μM each of F1 and F2, 0.76 μM each of C1, C2, R1, R2, D1 and D2, 1.53 μM each of CP1 and CP2), 2.2 μL (0.4 μM each of F1 and F2, 0.8 μM each of C1, C2, R1, R2, D1 and D2, 1.6 μM each of CP1 and CP2), 2.3 μL (0.42 μM each of F1 and F2, 0.84 μM each of C1, C2, R1, R2, D1 and D2, 1.67 μM each of CP1 and CP2). **(C)** Optimal reaction temperature for N gene detection. The reactions were performed under temperatures ranging from 62°C to 66°C at 1°C intervals. **(D)** Optimal primer concentration for N gene detection. Serial volumes of MCDA primer premixture (2.0 μL and 1.1 to 2.1 μL with 0.2 μL intervals) were used to prepare the reaction mixtures. Primer concentrations for serial volumes were as follows. 1.1 μL (0.22 μM each of F1 and F2, 0.44 μM each of C1, C2, R1, R2, D1 and D2, 0.88 μM each of CP1 and CP2), 1.3 μL (0.26 μM each of F1 and F2, 0.52 μM each of C1, C2, R1, R2, D1 and D2, 1.04 μM each of CP1 and CP2), 1.5 μL (0.3 μM each of F1 and F2, 0.6 μM each of C1, C2, R1, R2, D1 and D2, 1.2 μM each of CP1 and CP2), 1.7 μL (0.34 μM each of F1 and F2, 0.68 μM each of C1, C2, R1, R2, D1 and D2, 1.36 μM each of CP1 and CP2), 1.9 μL (0.38 μM each of F1 and F2, 0.76 μM each of C1, C2, R1, R2, D1 and D2, 1.52 μM each of CP1 and CP2), 2.0 μL (0.4 μM each of F1 and F2, 0.8 μM each of C1, C2, R1, R2, D1 and D2, 1.6 μM each of CP1 and CP2), 2.1 μL (0.42 μM each of F1 and F2, 0.84 μM each of C1, C2, R1, R2, D1 and D2, 1.68 μM each of CP1 and CP2). Ultrapure water served as negative control (NC).

The optimal amplification temperature confirmed by real-time MCTOP assay was determined at different temperatures ranging from 62°C to 66°C (with 1°C interval), and each experiment was repeated three times. The optimum temperature for MCTOP assay was 64°C, as a higher amplification rate and stronger fluorescence signals were observed at this temperature compared to the others (Figures 3A,C). In this study, the reaction temperature of 64°C was recommended as the optimum temperature for subsequent MCTOP based reaction. Additionally, the optimal primer concentration was determined using serial volumes of the MCDA primer premixture (2.2 μL and 1.1 to 2.3 μL with 0.2 μL intervals for ORF1ab- real-time MCTOP assay, 2.0 μL and 1.1 to 2.1 μL with 0.2 μL intervals for N- real-time MCTOP assay). The optimal primer concentrations for ORF1ab- and N- real-time MCTOP assays were 2.2 μL and 2.0 μL, respectively, as higher amplification rates and stronger fluorescence signals were observed at these optimal concentrations compared to the other concentrations (Figures 3B,D). In this study, the reaction temperature of 64°C was recommended as an optimum temperature, and N- primer volume of 2.0 μL and ORF1ab- primer volume of 2.2 μL were recommended as optimum primer concentrations for the rest MCTOP based reaction.

### Sensitivity, specificity and inhibitor tolerance test of COVID-19 MCTOP assay

**Figure 4:**
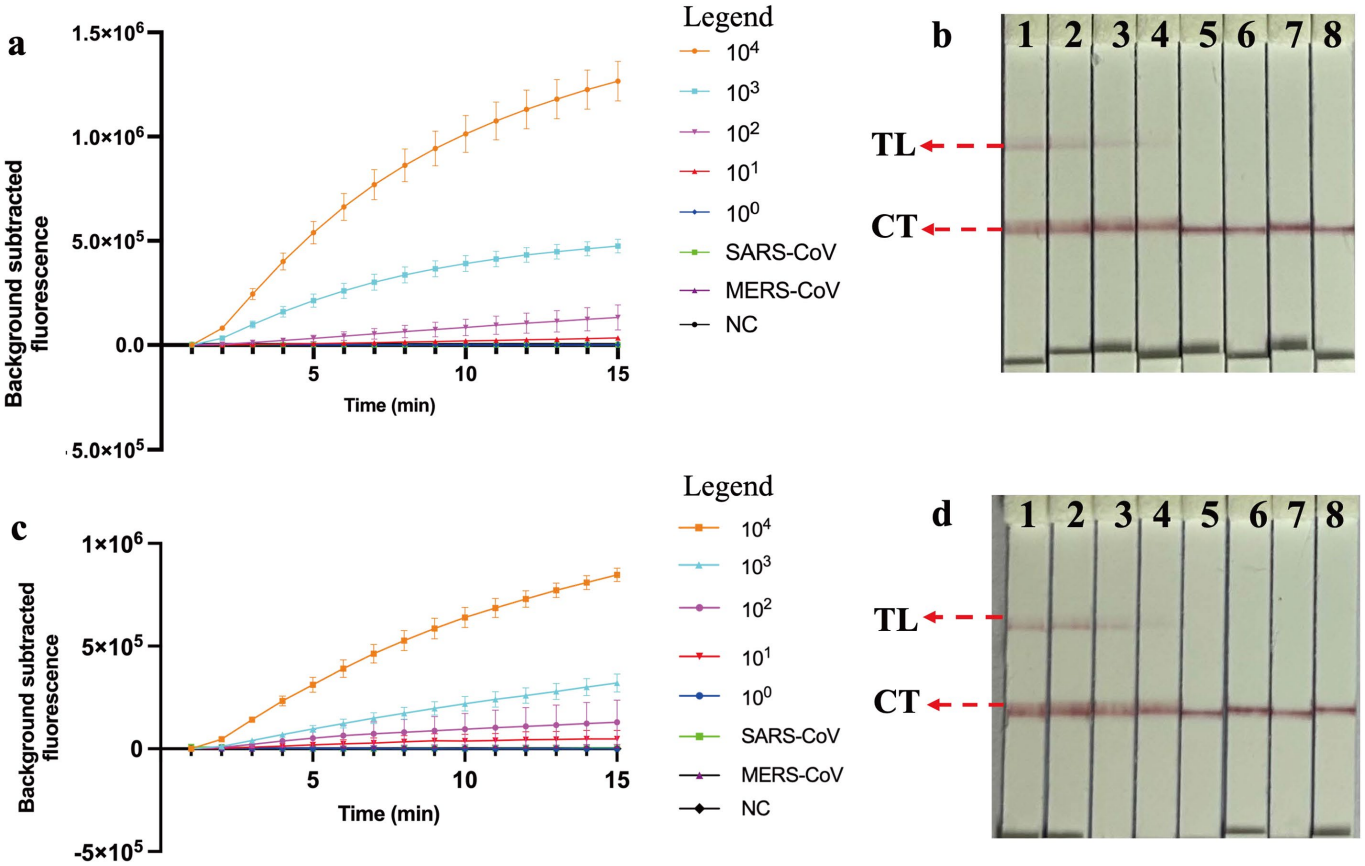
Sensitivity and specificity analysis of COVID-19 MCTOP assay. **(A,C)** COVID-19 MCTOP results were reported by real-time fluorescence **(A)** for ORF1ab-MCTOP result, and **(C)** for N-MCTOP result. Error bars represent the means ± standard error of means (SEM) from three replicates. **(B,D)** COVID-19 results were reported by lateral flow biosensor **(B)** for ORF1ab-MCTOP result, and **(D)** for NP-MCTOP result. Signals **(A)** /Biosensors **(B)** 1–8 represented the results of corresponding ORF1ab-plasmid levels of SARS-CoV-2 from 1 × 10^4^ to 1 × 10^0^ copies, and 1 × 10^4^ copies ORF1ab-plasmid of SARS-CoV and MERS-CoV, respectively, and ultrapure water served as negative control (NC). Signals **(C)** /Biosensors **(D)** 1–8 represented the results of corresponding N-plasmid levels of SARS-CoV-2 from 1 × 10^4^ to 1 × 10^0^ copies, and 1 × 10^4^ copies N-plasmid of SARS-CoV and MERS-CoV respectively, and ultrapure water served as negative control (NC). Control line (CL), Test line (TL).

The nucleic acid amplification curves were observed in the real-time fluorescent COVID-19 MCTOP assay, with template concentrations of ORF1ab- and N- plasmid varying from 1 × 10^4^ to 1 × 10^0^ copies (Figures 4A,C). The lowest detection limit of the real-time fluorescent COVID-19 MCTOP assay was 10 copies of ORF1ab- and N-plasmid templates, which was consistent with the lateral flow COVID-19 MCTOP method (Figures 4B,D). Taken together, the COVID-19 MCTOP assay achieved a sensitivity of 10 copies for each of target template.

The ORF1ab- and N-plasmid of SARS-CoV and MERS-CoV were subjected to evaluate the specificity of the COVID-19 MCTOP assay. The test results, visualized by the fluorescent COVID-19 MCTOP assay (Figures 4A,C) and lateral flow COVID-19 MCTOP method (Figures 4B,D) were the same, that was, the positive results were observed in ORF1ab- and N-plasmid of SARS-CoV-2 templates, while negative results in ORF1ab- and N-plasmid of SARS-CoV and MERS-CoV. The COVID-19 MCTOP methods, including both fluorescent and lateral flow COVID-19 MCTOP assays, successfully distinguished SARS-CoV-2 ORF1ab- and N-plasmid templates without cross-reactivity with either SARS-CoV or MERS-CoV templates, demonstrating high specificity for SARS-CoV-2 detection.

**Figure 5:**
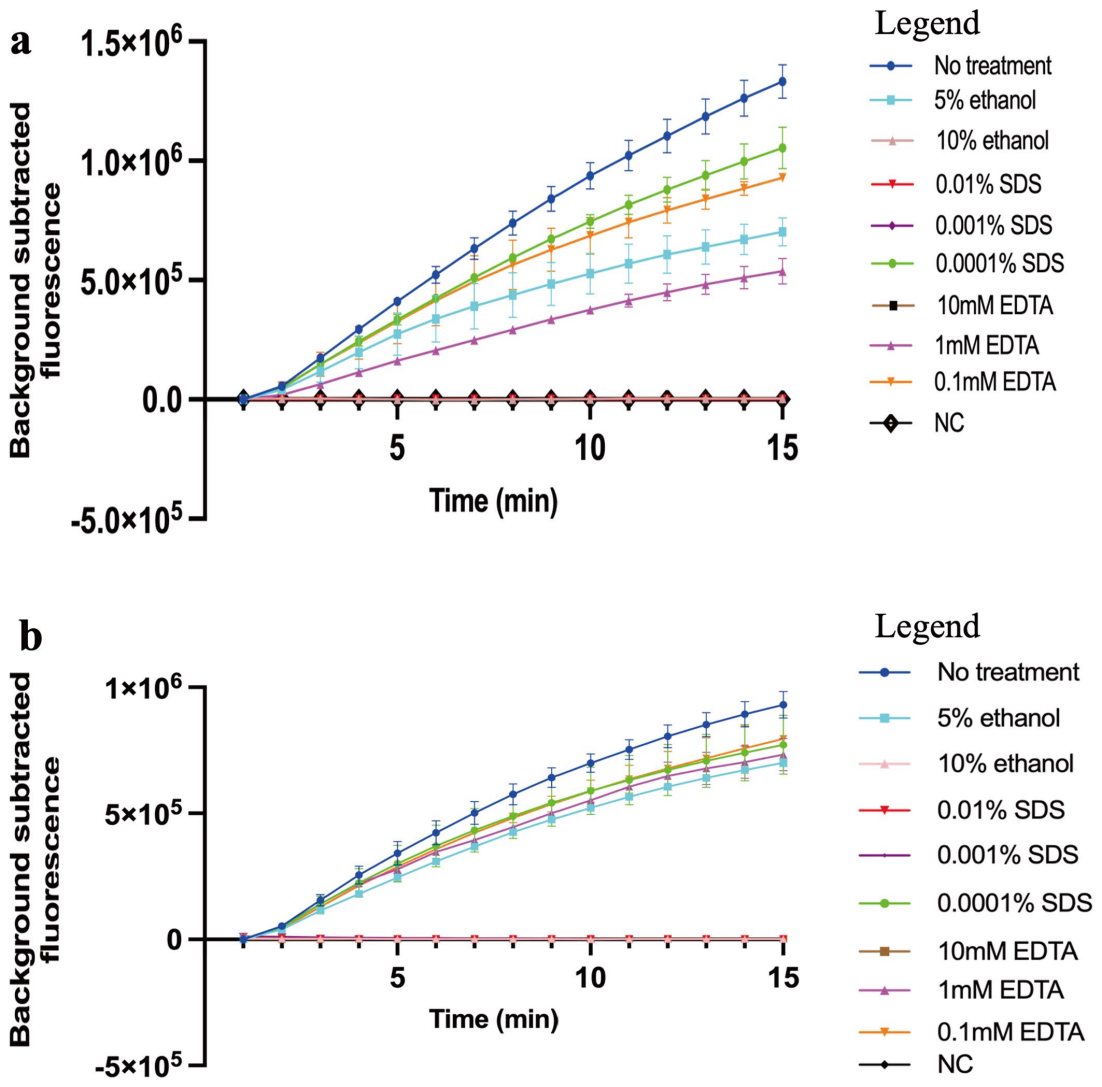
The effects of reaction inhibitors on COVID-19 MCTOP assay. 1 × 10^4^ copies of SARS-CoV-2 plasmid were used in the tolerance test. **(A)** For ORF1ab-MCTOP result, **(B)** for N-MCTOP result. Ultrapure water served as negative control (NC). Error bars represent the means ± standard error of means (SEM) from at three replicates.

In our study, 0.0001% SDS, 5% ethanol, and 1 mM and 0.1 mM EDTA slightly inhibited the MCTOP reaction, whereas 0.01 and 0.001% SDS, 10% ethanol, and 10 mM EDTA completely inhibited the reaction. Thus, COVID-19 MCTOP methods exhibit a certain inhibitors tolerance (Figure 5).

### Applicability of COVID-19 MCTOP method to clinical samples

**Figure 6:**
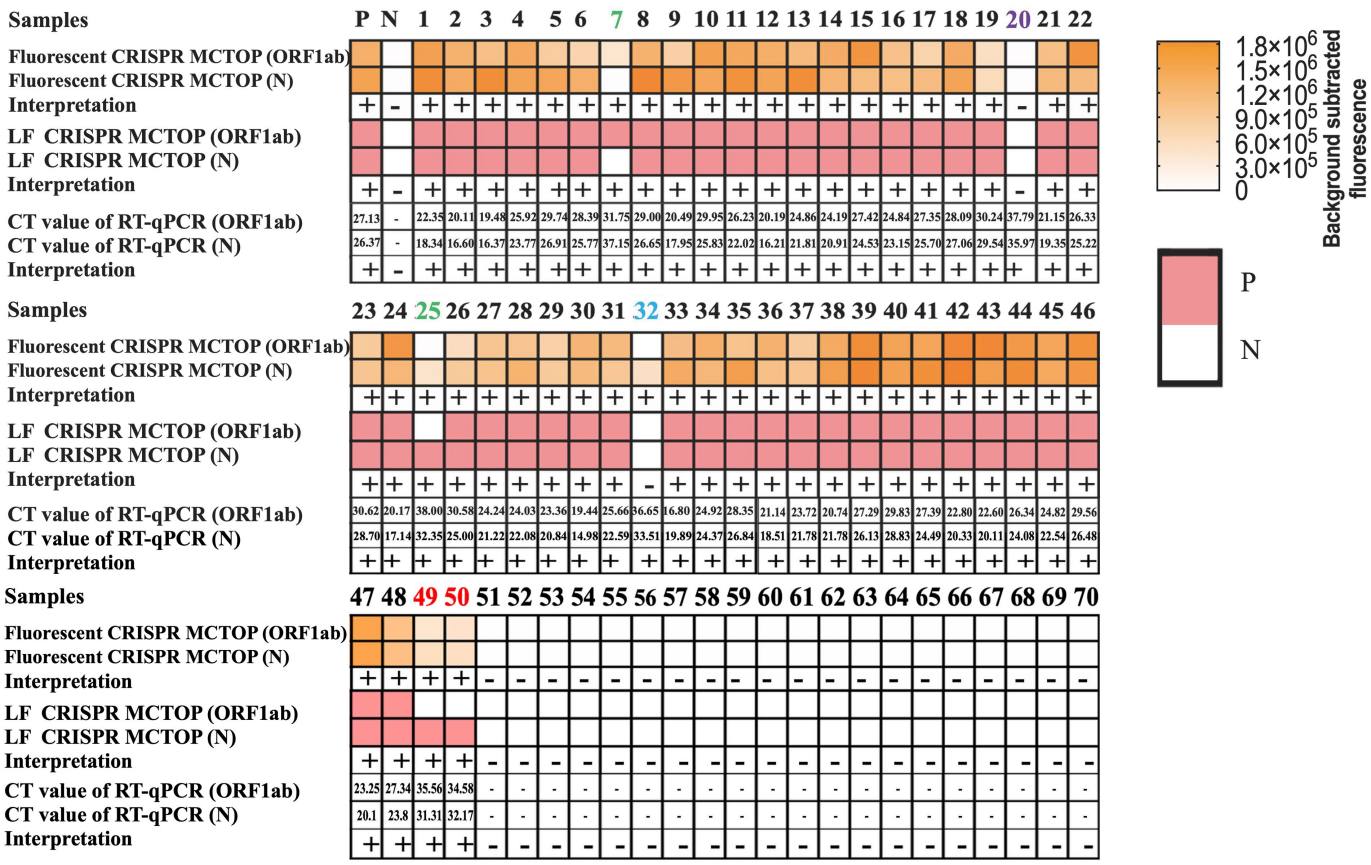
The results of COVID-19 MCTOP for clinical samples. In 70 clinical samples, SARS-CoV-2 were detected by fluorescence COVID-19 MCTOP and lateral flow COVID-19 MCTOP. MCTOP heat-maps represent the fluorescence values that subtracted the background (First three rows) or lateral flow biosensor qualitative test results (Middle three rows). RT-qPCR results are presented as Ct values (Last three rows). Among RT-qPCR-positive samples, those samples with single gene-positive results of one of COVID-19 MCTOP methods are marked with red numbers; those with single gene-positive results of both of COVID-19 MCTOP methods are marked with green numbers, that with one gene-positive for one of COVID-19 MCTOP and both gene-negative result for another COVID-19 MCTOP method are marked with blue numbers; and that with both gene-negative result of both of COVID-19 MCTOP methods are marked with purple numbers. P, positive; N, negative.

To verify the effectiveness of COVID-19 MCTOP method for clinic detection of SARS-CoV-2, the clinical samples of 70 pharyngeal swab were obtained, of which 50 samples were confirmed SARS-CoV-2 nucleic acid positive and 20 were negative. The test results from the fluorescence COVID-19 MCTOP and lateral flow COVID-19 MCTOP assays were similar, with 49 (98%) of 50 confirmed samples testing positive in the former method and 48 (96%) in the latter assay (Figure 6). In addition, no positive amplification was observed in negative samples by COVID-19 MCTOP assay (Figure 6). These results revealed that the proposed COVID-19 MCTOP method had a comparable sensitivity and specificity for diagnosis of SARS-CoV-2 infection compared to RT-qPCR (Supplementary Table S1).

## Discussion

In recent years, the CRISPR-Cas technology has emerged as a potential tool for nucleic acid detection with the advantages of rapidity, simplicity, versatility, ultrahigh sensitive, and specificity (Chertow, 2018). The combination of CRISPR-Cas technology with isothermal amplification methods such as loop-mediated isothermal amplification (LAMP), recombinase polymerase amplification (RPA), and MCDA, enables ultra-sensitive detection of nucleic acids. Among these isothermal amplification methods, MCDA is similar to LAMP in that it uses a DNA polymerase with strand displacement activity to achieve nucleic acid amplification at a constant temperature, characterized by fast amplification speed, high sensitivity, and strong specificity. Unlike the LAMP assay, which typically consists of 4–6 primers, MCDA can use up to 10 primers. Therefore, when applied to the detection of pathogens, including SARS-CoV-2, MCDA demonstrates higher sensitivity and speed compared to LAMP. This indicates that MCDA has similar application potential as LAMP but with enhanced sensitivity and speed (Wang et al., 2015; Zhang et al., 2020; Luu et al., 2021). Here, we propose a detection method called MCTOP, which combines MCDA with CRISPR-Cas technology for the detection of SARS-CoV-2.

The type V-B CRISPR proteins Cas12b (C2c1) from *Alicyclobacillus acidophilus* have RNA-guided endonuclease activity for recognition and cleavage of targeted nucleic acid sequences at the temperatures from 35°C to 65°C (Teng et al., 2018). Accordingly, AapCas12b enzyme is applicable for isothermal amplification that requires a relatively high amplification temperature due to its resistance to higher temperatures (Joung et al., 2020). The compatibility of MCDA and CRISPR-Cas12b in terms of reaction temperature (60°C to 65°C) enables the combination of the two techniques, which has the potential for POCT. In this context, MCTOP method, which includes CRISPR-Cas12b and MCDA, is designed to detect SARS-CoV-2 by one-step process (Figure 1), with the advantages of efficiency and simplicity. The MCDA assay was performed using PAM site modification primers (CP1 or R1). Then, the MCDA products will be incorporated with PAM sites, which are used for Cas12b/gRNA complex-associated recognition and cleavage. In principle, the proposed method can also detect target sequences without PAM sites if the target sequences fulfill the requirements of MCDA design. In MCTOP assay, the fluorescence signals and lateral-flow readouts can be used to analyze the amplified signals, which improves the ability to detect pathogens. Compared with other studies of CRISPR-based SARS-CoV-2 tested platforms (Broughton et al., 2020; Huang et al., 2020; Zhu et al., 2021), MCTOP assay requires only simple apparatus for MCDA-mediated isothermal amplification and one liquid-handling step to minimize the risk of cross-contamination between tested samples. This method is suitable for use in point-of-care and field laboratories due to its easy operation and without expensive instruments.

The COVID-19 MCTOP assay used to assess ORF1ab- and N-plasmid showed a promising detection limit of 10 copies under the optimal reaction conditions (Figure 3), which is similar to the other researches (Ali et al., 2020; Chen et al., 2020; Li et al., 2021). It could be attributed to the excellent sensitivity of COVID-19 MCTOP, as the resultant cDNA from reverse transcription was used as the template for subsequent amplification. The whole process for fluorescence COVID-19 MCTOP takes 70 min, including 15 min for nucleic acid preparation, and 55 min for fluorescence MCTOP reaction. For lateral flow COVID-19 MCTOP assay, the result interpretation takes less than 5 min, in addition to the processes mentioned above. The lateral flow COVID-19 MCTOP assay can detect OFR1ab- and N-plasmid with 10 copies limit of detection, which is consistent with fluorescence COVID-19 test (Figure 3).

In the specificity analysis, the COVID-19 MCTOP assay showed 100% specificity (Figure 3), with a positive signal identified only in SARS-CoV-2-plasmid templates, but not in SARS-CoV or MERS-CoV plasmid templates. In the MCTOP system, two rounds of targeted gene detection were performed to ensure the accuracy of the results. Firstly, targeted genes were specifically amplified by MCDA assay in the first-round detection (Figure 1, Step2). Then, the MCDA products were detected by CRISPR-Cas-mediated sequence-specific detection in the second-round detection, which utilizes gRNA to guide the Cas12b nuclease for sequence-specific recognition and cleavage (Figure 1, Step3). Hence, the MCTOP assay proposed in this study is an accurate and reliable tool for SARS-CoV-2 nucleic acid detection.

The MCTOP method established in this study is a qualitative detection method, which makes it impossible to calculate statistical data such as the coefficient of variation (CV), standard deviation (SD), mean values, random error (RE), and systematic error (SE) to determine method precision, accuracy, and total allowable error (TEa) for the statistical validation of the MCTOP method. Therefore, we further compared the sensitivity and specificity of our method with RT-qPCR to assess the diagnostic performance of the MCTOP method (Florkowski, 2008). The 70 pharyngeal swab samples, including 50 samples from COVID-19 patients and 20 samples from non-COVID-19 individuals, were used for the clinical verification of COVID-19 MCTOP assay (Figure 5; Supplementary Table S1). Positive signals were not observed in 20 RT-qPCR negative samples tested by COVID-19 MCTOP tests, confirming its high specificity in clinical applications. Positive signals were observed in RT-qPCR positive samples by fluorescence COVID-19 MCTOP (49/50, 98%) and lateral flow COVID-19 MCTOP assays (48/50, 96%). In comparison to RT-qPCR, fluorescence COVID-19 MCTOP exhibits comparable sensitivity and can detect clinical samples with a Ct value below 35.97. This is probably due to SARS-CoV-2 viral load in those samples (with a Ct value above 35.97) was lower than detection limit of COVID-19 MCTOP. When the COVID-19 MCTOP assay was used for clinical sample detection, we suggest enlarging template volume to 5 μL to enhance the sensitivity. When testing clinical samples, our positive detection rate was relatively high compared to the other method (Li et al., 2021). Consequently, our method holds promise for rapid screening of SARS-CoV-2 infection, thereby contributing to the containment of SARS-CoV-2 transmission. Our research showed that MCTOP assay can be used for clinical samples, but more types of clinical samples are needed to validate the applicability of MCTOP assay in further study. Moreover, the MCTOP assay also has the technical advantage of isothermal amplification. It is insensitive to PCR common inhibitors, can tolerate various inhibition of nucleic acids, and is less affected by large amounts of salt ions in sample buffer (Kaneko et al., 2007; Li et al., 2021). Due to the high mutation rate of SARS-CoV-2, we compared the primers and gRNA sequences of our method against the epidemic mutant strains present at the time of method establishment and the current epidemic strains published by the WHO. We found that the gRNA sequences were consistent across different mutant strains. Additionally, the ORF1ab and N gene primer sequences were highly conserved, with only a few variants showing 1–2 base pair differences among 1–2 primers (Supplementary Figure S1). This indicates that the MCTOP method is applicable to different mutant strains.

Currently, COVID-19 swab tests for home use mainly include nucleic acid-based and antigen-based testing methods (Drain, 2022). Among these, antigen rapid diagnostic tests (Ag-RDTs) using nasal swabs are increasingly being utilized worldwide for the screening and diagnosis of COVID-19. However, the analytical sensitivity of Ag-RDTs and some rapid molecular tests is lower than that of most RT-qPCR tests. Ag-RDTs exhibit relatively low sensitivity (44–84.3%) in detecting infected individuals at any stage of infection, and they are not effective in identifying presumably infectious individuals (Filgueiras et al., 2023; Sugiharto et al., 2023; Viloria Winnett et al., 2023). Thus, it is not suitable for border COVID-19 testing, as it is prone to missing cases. The obtained results demonstrated that MCTOP is an ultrasensitive analytical technique for SARS-CoV-2 detection. In lateral flow COVID-19 MCTOP assay, biosensors are easy to use and do not require specific training or equipment, making it suitable for on-site, field, and clinical detection, especially in poor areas. Moreover, lateral flow COVID-19 MCTOP has the potential to be further developed into a small self-testing instrument for home-based screening.

The lateral flow COVID-19 MCTOP method requires opening the reaction tube during result interpretation phase, which can generate aerosols containing high concentrations of COVID-19 MCTOP amplicons. Given that the COVID-19 MCTOP is an ultra-sensitive detection method, repeated use of the lateral flow COVID-19 MCTOP in the same laboratory could lead to carryover contamination through contaminated pipettes, the skin of personnel, or workspace surfaces. Therefore, to prevent aerosol contamination from confounding test results, laboratories performing lateral flow COVID-19 MCTOP tests should conduct the biosensor result interpretation phase in a separate area and adopt cautious preventive measures. The decontamination process, once contamination occurs, is both time-consuming and costly. Additionally, the CRISPR reagent pre-added at the top of the reaction tube in this study may slide down and mix with the amplification system before the amplification is complete, thereby affecting the accuracy of the detection. Therefore, it is crucial to carefully pre-add the CRISPR reagent at the top of the reaction tube. In future applications, if a specially designed reaction tube can be developed, it would help avoid the aforementioned situation and improve the accuracy of the detection.

## Conclusion

This study successfully developed a CRISPR-Cas-mediated detection platform (MCTOP, *M*CDA-*C*RISPR-Cas12b-based *t*esting in *o*ne-*p*ot) targeting ORF1ab and N genes of SARS-CoV-2, which combines MCDA isothermal amplification and CRISPR-Cas-mediated sequence-specific detection into a one-pot reaction. The proposed platform has been initially validated using standard plasmids and clinical samples, and the results can be visualized by the real-time fluorescence instrument and lateral flow biosensor. Preliminary results show that COVID-19 MCTOP assay is rapid, convenient, and exhibits comparable sensitivity and strong specificity for SARS-CoV-2 detection. Moreover, it has potential utility for clinical diagnosis and epidemiologic surveillance of SARS-CoV-2 infections, especially in developing countries, where no complicated equipment or experienced technicians required. Schematically, MCTOP can be amplified for detection of various sequences simply by designing the appropriate primers and gRNAs.

## Data Availability

The original contributions presented in the study are included in the article/Supplementary material, further inquiries can be directed to the corresponding author.

## Footnotes

^1^ http://primerexplorer.jp/e/

^2^ http://www.ncbi.nlm.nih.gov/tools/primer-blast/
