# Supplementary material for: One-pot MCDA-CRISPR-Cas-based detection platform for point-of-care testing of severe acute respiratory syndrome coronavirus 2
Source: Front Microbiol. 2024 Dec 6;15:1503356. doi: 10.3389/fmicb.2024.1503356 (PMC11659237; doi:10.3389/fmicb.2024.1503356)
Supplement: Supplementary file 1 [file Table_1.DOCX]

**Supplementary files**

**Table S1** Comparison of MCTOP and RT-qPCR methods for the detection of COVID-19 in clinical samples

| **Detection methods** |  | **COVID-19 patients** | **Non-COVID-19 patients** | **Total** |
| --- | --- | --- | --- | --- |
| Fluorescence COVID-19 MCTOP | Number of positive | 49 | 0 | 49 |
|  | Number of negative | 1 | 20 | 21 |
|  | Total | 50 | 20 | 70 |
|  | Sensitivity | 98% | | |
|  | Specificity | 100% | | |
| Lateral flow COVID-19 MCTOP | Number of positive | 48 | 0 | 48 |
|  | Number of negative | 2 | 20 | 22 |
|  | Total | 50 | 20 | 70 |
|  | Sensitivity | 96% | | |
|  | Specificity | 100% | | |
| RT-qPCR | Number of positive | 50 | 0 | 50 |
|  | Number of negative | 0 | 20 | 20 |
|  | Total | 50 | 20 | 70 |
|  | Sensitivity | 100% | | |
|  | Specificity | 100% | | |


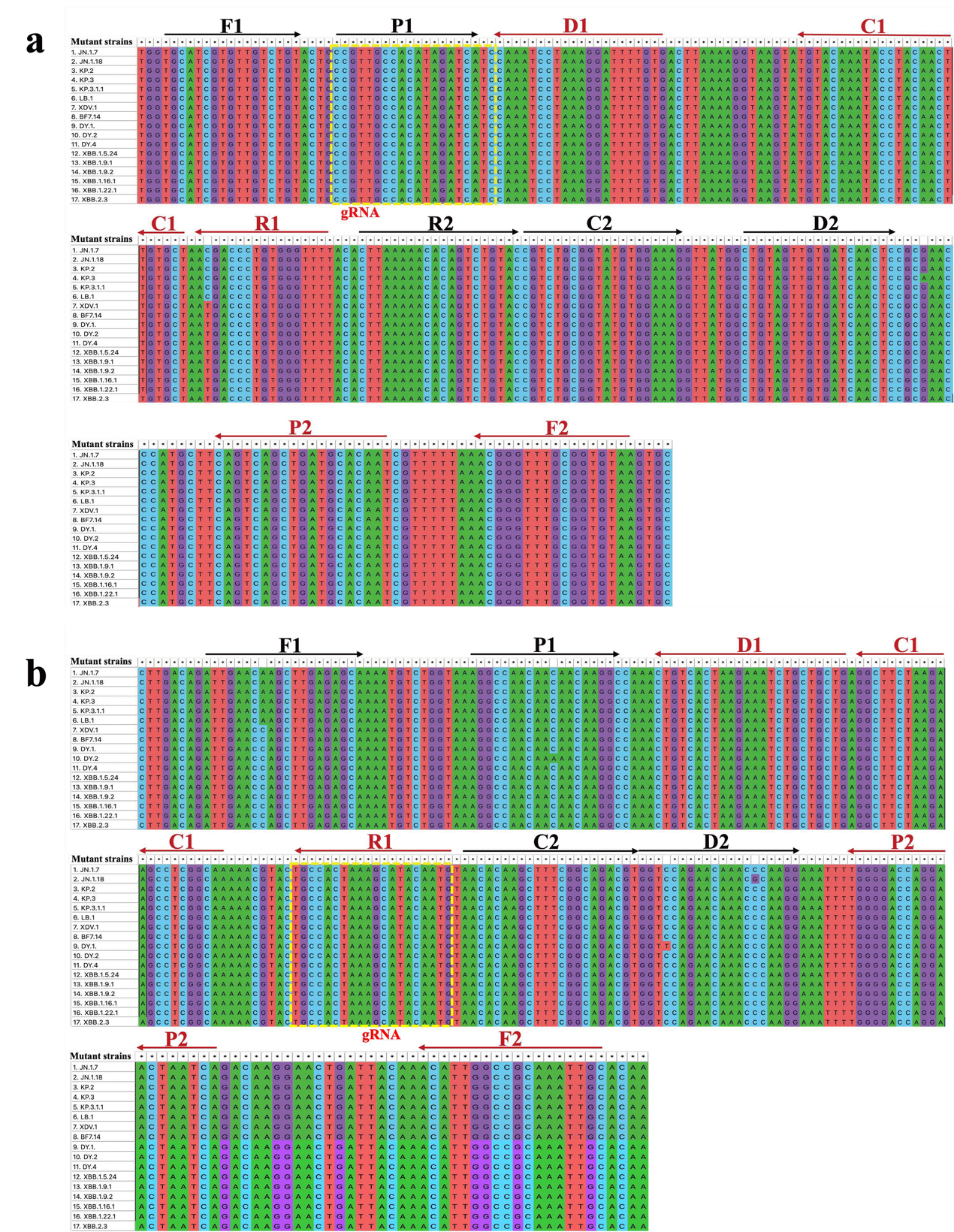


**Figure S1. Comparison sequences between SARS-CoV-2 variant strains at the sites targeted by the primers and gRNA used in this report.** The primer sets and gRNA target different regions in the ORF1ab (a) and N (b) genes of the SARS-CoV-2 genome. The sites of primer sequence were indicated in arrows and gRNA are boxed in yellow.
